# Dermoscopy of Cutaneous Melanoma Metastases: A Comprehensive Literature Review

**DOI:** 10.3390/diagnostics16050738

**Published:** 2026-03-02

**Authors:** Martina D’Onghia, Serena Agueci, Biagio Scotti, Francesca Falcinelli, Sofia Lo Conte, Alessandra Cartocci, Christian Dorado Cortez, Emi Dika, Linda Tognetti, Pietro Rubegni, JeanLuc Perrot, Elisa Cinotti

**Affiliations:** 1 Dermatology Unit, Department of Medical, Surgical and Neurological Sciences, University of Siena, 51300 Siena, Italy; serena.agueci@gmail.com (S.A.); f.falcinelli@student.unisi.it (F.F.); sofia.loconte@student.unisi.it (S.L.C.); alessandra.cartocci@dbm.unisi.it (A.C.); linda.tognetti@dbm.unisi.it (L.T.); pietro.rubegni@unisi.it (P.R.); elisa.cinotti@unisi.it (E.C.); 2 Department of Medical and Surgical Sciences, Alma Mater Studiorum, University of Bologna, 40126 Bologna, Italy; biagio.scotti2@unibo.it (B.S.); emi.dika3@unibo.it (E.D.); 3 Dermatology Unit, IRCCS Azienda Ospedaliero-Universitaria di Bologna, 40138 Bologna, Italy; 4 Dermatology Department, Centre Hospitalier Universitaire de Saint-Étienne, 42270 Saint-Etienne, Francej.luc.perrot@chu-st-etienne.fr (J.P.); 5 Melanoma Unit, University Hospital of Saint-Etienne, 42270 Saint-Etienne, France

**Keywords:** metastases, melanoma, dermoscopy, review

## Abstract

**Background**: Cutaneous melanoma metastases (CMM) represent a clinically relevant manifestation of advanced melanoma and may constitute the first sign of disseminated disease. Their diagnosis is challenging because CMM shows highly variable clinical and dermoscopic presentations and frequently mimic other benign or malignant skin lesions. Although dermoscopy is routinely used to improve skin lesion assessment, dermoscopic criteria specific to CMM remain poorly defined and still non-standardized. **Methods**: We performed a narrative review of the literature to summarize dermoscopic features reported in CMM. MedLine (via PubMed) and Web of Science were searched up to 3 December 2025 using the keywords “dermoscopy” and “melanoma metastasis,” complemented by manual reference screening. Eligible studies were English-language full-text articles in peer-reviewed journals providing a complete dermoscopic description. Extracted data included patient demographics and major dermoscopic criteria, categorized as global patterns and focal dermoscopic and vascular structures. Due to heterogeneity, results were synthesized descriptively. **Results**: Twenty studies were included, comprising 774 patients. Dermoscopic findings were markedly heterogeneous. Globally, lesions frequently showed homogeneous pigmentation with variable colors and included amelanotic presentations. Commonly evaluated focal features included irregular dots and globules, crystalline structures, peripheral gray dots, and lacuna-like areas. Vascular patterns were prominent, particularly serpentine and corkscrew-like vessels. **Conclusions**: CMM dermoscopy is characterized by substantial heterogeneity and a lack of standardized criteria. Systematic classification of recurring dermoscopic features may improve diagnostic consistency and provide an interpretable framework for future artificial intelligence-based approaches supporting non-invasive recognition of melanoma metastases.

## 1. Introduction

Melanoma represents one of the most aggressive skin cancers, with a significantly rising incidence worldwide [1]. Advanced stages are associated with high mortality, largely due to its metastatic potential and often unpredictable dissemination patterns [2]. Beyond lymph nodes and visceral organs, melanoma frequently metastasizes to the skin [3,4].

Remarkably, melanoma is well known for delayed cutaneous metastases (CMM), which can occur even after years of stable disease. In 10–30% of cases, secondary cutaneous involvement represents the first sign of disseminated melanoma [5,6], and patients with cutaneous metastases generally face a poor prognosis, with a median melanoma-specific survival of approximately 5.07 years and a 5-year survival rate of 52% [7]. Consequently, early recognition of CMM is essential for optimal patient management.

However, the diagnosis of CMM is often challenging due to their highly polymorphic presentation, which encompasses a broad spectrum of both clinical and dermoscopic features, mimicking other benign or malignant skin conditions [8]. Indeed, secondary skin involvement may appear as red, pink, skin-colored, bluish, or pigmented papules, nodules, plaques, or ulcers, occurring either as solitary or multiple lesions [9].

Dermoscopy currently represents an invaluable, non-invasive, and easily applicable diagnostic tool that is routinely employed in clinical practice [10]. It facilitates the differentiation of primary melanoma from benign lesions, enhancing diagnostic accuracy and precision compared with naked-eye examination alone, although it has inherent limitations in magnification and depth of visualization [11]. By providing detailed visualization of subsurface structures, dermoscopy allows clinicians to identify subtle features of melanoma at an earlier stage, thereby supporting early diagnosis and improving patient management [12].

In addition, the growing availability of digital dermoscopic data has recently stimulated interest in advanced analytical approaches, including artificial intelligence-based models, which may further expand the diagnostic and prognostic potential of dermoscopy in melanoma management [13].

Nevertheless, the dermoscopic features of CMM remain poorly characterized, and no standardized criteria are available to distinguish them from other benign or malignant skin lesions. In this context, we present a narrative review of the dermoscopic characteristics of CMM, aiming to identify reproducible patterns that may enhance diagnostic accuracy and aid clinicians in differential diagnosis.

## 2. Materials and Methods

The primary aim of this narrative review was to provide a comprehensive overview of the dermoscopic features reported in studies focusing on CMM. A literature search was conducted using MedLine (via PubMed) and Web of Science (WOS) databases, covering all articles published up to 3 December 2025. The search strategy combined the keywords “dermoscopy” and “melanoma metastasis.”

In addition, the reference lists of selected articles were manually screened to identify further relevant studies. To be included in the final review, studies had to be full-text original articles published in international, peer-reviewed journals. Eligible study designs included prospective or retrospective cohort studies, cross-sectional studies, case series, case reports, and letters.

Studies were excluded if they did not provide a complete dermoscopic description of the reported lesions.

After study selection, data extraction was independently performed by two authors (S.A. and M.D.), while a third author (B.S.) reviewed the extracted data and resolved any discrepancies. For each included article reporting individual cases or case series of CMM, both general study characteristics and dermoscopic data were systematically collected, in accordance with previously published approaches in the literature on CMM dermoscopy. Specifically, the extracted variables included year of publication, number of reported CMM lesions, and demographic characteristics of the study population, including age and gender.

In addition, all dermoscopic findings described in the included studies were recorded, with particular emphasis on major dermoscopic criteria. These criteria were categorized into global dermoscopic patterns and focal dermoscopic and vascular structures, and for each study, the number of lesions in which each dermoscopic criterion was reported to be present was also documented.

Given the heterogeneity of the included studies, data synthesis primarily focused on summarizing the frequency with which each dermoscopic criterion was used by the authors to evaluate the lesions in their respective studies, rather than on quantitative pooling of results. A dermoscopic criterion was considered present only when it was explicitly described by the original study authors.

## 3. Results

### 3.1. Literature Review

In this literature review, we identified 20 studies investigating the dermoscopic features of CMM [5,8,9,10,11,14,15,16,17,18,19,20,21,22,23,24,25,26,27,28].

Overall, these studies included 774 patients, of whom 44% were female. The reported mean age at primary melanoma diagnosis varied substantially, ranging from 31 years [5] to 88 years [20] (Supplementary Table S1). Across the included cohorts, dermoscopic features focused on both global and focal aspects, as well as the vascular patterns characteristic of CMM lesions.

### 3.2. Global Dermoscopic Features and Pigmentation Patterns

All global dermoscopic criteria are summarized in Table 1. Regarding general dermoscopic features, five authors described cases of amelanotic lesions [5,15,17,25,26].

Nevus-like patterns were evaluated in three studies [9,23,25], while two studies documented a saccular pattern [25,26]. Ulceration or crusting was rarely reported, with Jaimes et al. [17] documenting this feature in 11 lesions.

With respect to pigmentation, a predominantly homogeneous pigmentation pattern was generally noted. Seven studies evaluated the presence of brown or black pigmentation [5,8,9,11,16,22,24], while blue pigmentation was also frequently reported [5,8,9,11,19,21,25]. Pink or red [5,8,9], gray [5,9,19,25], and whitish coloration [9,10,14] were less consistently assessed.

### 3.3. Focal Dermoscopic Features

Among focal dermoscopic features, irregularly distributed black dots or globules were the most frequently evaluated criteria [8,9,11,14,22,24,25] (Table 1). Crystalline structures [8,21], peripheral gray dots [5,8,21,25], and lacuna-like areas [8,10,11] were also reported. A light brown halo and peripheral erythema were described by three authors [5,8,25]. Less common findings included streaks [8,9], microhemorrhages [8], and inverse network patterns [22,25].

### 3.4. Dermoscopic Features of Vascular Structures

Serpentine vessels were the most frequently evaluated vascular pattern [8,9,10,17]. Corkscrew-like vessels [5,8,9,11,17,19,26], linear vessels [8,9,14,18,26], and dotted vessels [8,9,17,25] were also described (Table 2).

## 4. Discussion

This narrative review summarizes the available evidence on the dermoscopic features of CMM, highlighting the wide morphological variability of these lesions and the absence of standardized diagnostic criteria. Overall, the included studies consistently demonstrate that CMM may display a broad spectrum of dermoscopic presentations, frequently overlapping with benign melanocytic lesions, primary melanoma, and other malignant skin tumors [5,8,9,10,11,14,15,16,17,18,19,20,21,22,23,24,25,26,27,28]. This marked heterogeneity represents a major diagnostic challenge in daily clinical practice and may partly explain why dermoscopic criteria specific for CMM have not yet been formally codified.

**Figure 1:**
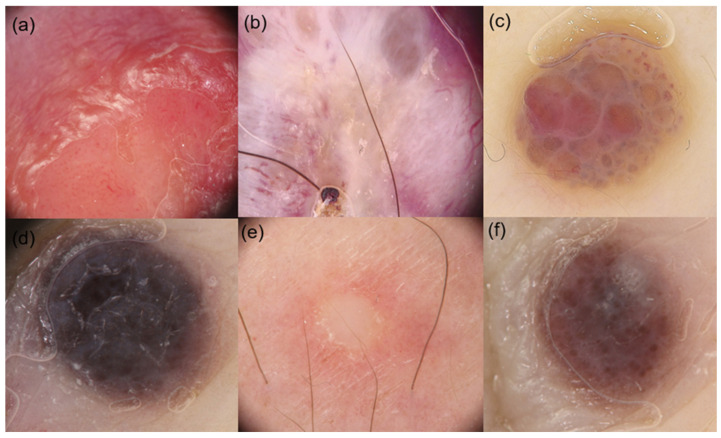
Global and focal dermoscopic features of CMM. (**a**) Vascular-like pattern, red-orange pigmentation, arborizing and dotted vessels; (**b**) Polymorphic pattern, crystalline structures, arborizing and serpentine vessels; (**c**) Angioma-like pattern, pink-purple pigmentation, light brown halo, red or purplish pseudo-lacunae, arborizing vessels; (**d**) Blue nevus-like pattern, blue-brown pigmentation, light brown halo; (**e**) Amelanotic pattern, white-yellowish pigmentation, peripheral erythema; (**f**) Nevus-like globular pattern, blue-brown pigmentation.

**Figure 2:**
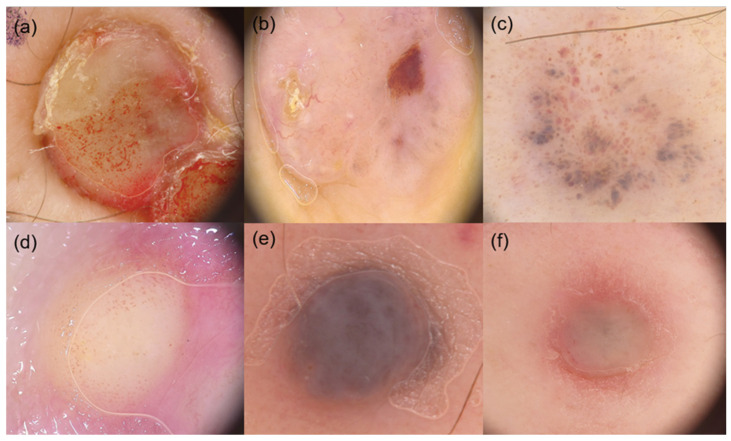
Global and focal dermoscopic features of CMM. (**a**) Vascular-like pattern, red-orange pigmentation, comma vessels; (**b**) Polymorphic pattern, pink-purple pigmentation, light brown halo, crystalline structures, arborizing and serpentine vessels; (**c**) Polymorphic pattern and brown globules; (**d**) Amelanotic pattern, white-yellowish pigmentation, peripheral erythema, glomerular vessels; (**e**) Angioma-like pattern, blue-brown pigmentation, red or purplish pseudo-lacunae; (**f**) Amelanotic pattern, yellow-whitish pigmentation, peripheral erythema, dotted vessels.

Regarding global dermoscopic features, most studies reported a predominantly homogeneous pattern with variable pigmentation, ranging from brown and black to blue, gray, pink, red, or whitish hues [5,9,11,14,16,19,21,22,24,25] (Figure 1 and Figure 2). Current literature shows that the blue pattern is a frequently reported feature in CMM, often resembling the appearance of blue nevi [9,10]. This finding is thought to reflect the dermal localization of metastases, rather than involvement of the dermoepidermal junction, as further supported by confocal microscopy studies [29].

Amelanotic or hypomelanotic lesions were described in several cohorts [5,15,17,25,26], confirming that lack of pigmentation is not uncommon in CMM and may further complicate both clinical and dermoscopic recognition. Nevus-like and saccular patterns were only sporadically evaluated [5,9,23,25,26], while ulceration or crusting was rarely reported [17], suggesting limited diagnostic utility of these features when assessed dermoscopically.

Finally, Costa et al. [11] reported a vascular-like and angioma-like pattern in CMM. Vascular structures are believed to reflect tumor-induced angiogenesis, a key driver of cancer progression and distant dissemination [30]. Interestingly, Jaimes et al. [17] reported that vascular patterns were more frequently associated with cutaneous metastatic melanoma originating from primary melanomas with a Breslow thickness of 2–4 mm. Notably, in CMM with angioma-like features, vessels were often visible within the well-defined lacunae, which may represent a useful diagnostic criterion for differentiating angioma-like CMM from benign angiomas.

Analysis of focal dermoscopic features further confirmed the polymorphic nature of CMM (Figure 1 and Figure 2). Irregularly distributed dots and globules represented the most frequently evaluated focal criteria [8,9,11,14,22,24,25], although their prevalence varied widely among studies. Additional focal structures, such as crystalline structures [8,21], peripheral gray dots [5,8,21,25], lacuna-like areas [8,10,11], and light brown halos or peripheral erythema [5,8,25], were inconsistently reported. Less common findings, including streaks [8,9], microhemorrhages [8], and inverse network patterns [22,25], were only sporadically described.

Collectively, these observations indicate that no single focal dermoscopic structure can be considered pathognomonic for CMM, and that diagnostic interpretation must rely on the overall morphological context. This absence of consistent surface structures may be attributed to the epidermal sparing typically observed in CMM, which histopathologically presents as dermal “bottom-heavy” infiltrates.

As already stated, vascular structures constituted another important component of dermoscopic evaluation. Serpentine vessels were the most frequently described vascular pattern across studies [8,9,10,17], followed by corkscrew-like vessels [5,8,9,11,17,19,26], as well as linear [8,9,14,18,25] and dotted vessels [8,9,17,25]. Several authors suggested that vascular patterns may be more prominent in CMM than in primary melanoma, possibly reflecting enhanced neoangiogenesis associated with metastatic progression [30,31]. Nevertheless, the considerable variability in vascular morphology and distribution limits their diagnostic specificity when considered in isolation.

Beyond melanoma-specific findings, several authors have analyzed the dermoscopic features of cutaneous metastases arising from systemic malignancies, highlighting both shared characteristics and tumor-specific differences [9,10,27]. Overall, cutaneous metastases from non-melanoma primaries tend to exhibit a predominantly structureless dermoscopic appearance, most often characterized by white, pink, or red coloration and a prominent vascular component, reflecting their dermal-based growth and limited epidermal involvement [9,10,27]. In contrast, melanoma metastases appear to represent a distinct subgroup, as they more frequently retain pigmentation-related features, including blue or blue-white coloration and, in some cases, melanocytic structures, as highlighted by the present review.

In addition, the growing availability of digitally acquired and annotated dermoscopic images has recently stimulated interest in advanced analytical approaches, including artificial intelligence-based models, which may further expand the diagnostic and prognostic potential of dermoscopy in melanoma management [13]. Emerging evidence suggests that AI systems may be capable not only of improving lesion classification but also of capturing subtle and complex dermoscopic patterns associated with melanoma behavior [32]. Importantly, the development and clinical reliability of such models are intrinsically dependent on the availability of well-defined, standardized, and biologically interpretable dermoscopic criteria. Moreover, where feasible, pairing dermoscopy with reflectance confocal microscopy (RCM) on the same lesion may further enhance AI-driven performance by providing complementary information across different skin depths, potentially maximizing algorithmic yield in lesions predominantly involving the superficial to mid dermis [33]. In this context, the systematic overview of dermoscopic features of CMM provided by the present review may represent a valuable knowledge base for future AI-driven applications aimed at supporting the recognition and characterization of melanoma metastases, including multimodal approaches integrating dermoscopy and RCM when accessible.

The main strength of this review lies in its comprehensive synthesis of the dermoscopic features of CMM reported across the literature, providing a structured overview of global patterns, focal structures, and vascular features in a field where evidence remains fragmented. By organizing and contextualizing heterogeneous data, this work highlights recurrent dermoscopic characteristics while acknowledging their variability, thereby offering clinically meaningful insights.

Nevertheless, several limitations must be considered. The majority of available studies are retrospective and based on case reports or small case series, resulting in substantial heterogeneity in study design, patient selection, and dermoscopic assessment. Dermoscopic criteria were not uniformly defined or systematically evaluated across studies, and many authors focused on selected features rather than adopting standardized assessment frameworks. In addition, key methodological details, such as the dermoscopic light modality (polarized vs. non-polarized) and clinically relevant variables (e.g., anatomical site, lesion size, and the presence of additional metastases), were often inconsistently reported or unavailable. This heterogeneity precluded quantitative synthesis and limits direct comparison across studies. Furthermore, observer-dependent interpretation of dermoscopic images and potential publication bias toward unusual presentations may have influenced the reported findings.

## 5. Conclusions

This narrative review contributes to the limited literature on cutaneous melanoma metastases by systematically summarizing dermoscopic features reported across published studies. The marked heterogeneity of CMM underscores the need for standardized assessment protocols and larger multicenter studies. Establishing a shared dermoscopic framework may improve non-invasive recognition of melanoma metastases and facilitate the development of artificial intelligence-based diagnostic tools.

## Figures and Tables

**Table 1:** Global and Focal Dermoscopic Features of CMM Lesions Reported in Included Studies.

			Global CMM Features										Focal CMM Features					
	N° of Lesions	Homogeneous Main Color Pigmentation						Amelanotic	Vascular	Saccular	Nevus Like Pattern *	Angioma-Like	Black Dots or Globules Irregular Distributed	Light Brown Halo	Peripheral Gray Dots	Crystalline Structures	Peripheral Erythema	Lacune Like Areas
		Brown or Gray	Brown or Black	Whitish or Gray	Blue	Pink or Red	Two or More Colors											
Pizzichetta et al., [23]	4	-	-	-	-	-	1	-	-	-	3	-	-	-	-	-	-	-
Bono et al., [5]	130	51	21	-	16	42	5	42	-	-	-	-	-	53	33	-	16	-
Virgili et al., [14]	1	-	-	1	-	-	-	-	-	-	-	-	1	-	-	-	-	-
Minagawa et al., [15]	1	-	-	-	-	-	-	1	-	-	-	-	-	-	-	-	-	-
Contreras-Steyls et al., [16]	1	-	1	-	-	-	-	-	-	-	-	-	-	-	-	-	-	-
Jaimes et al., [17]	47	-	-	-	-	-	-	47	-	-	-	-	-	-	-	-	-	-
Costa et al., [11]	71	-	11	-	22	-	-	-	14	-	-	9	15	-	-	-	-	8
Duman et al., [18]	1	-	-	-	-	-	1	-	-	-	-	-	-	-	-	-	-	-
Hoelt et al., [19]	3	1	-	-	1	-	1	-	-	-	-	-	-	-	-	-	-	-
Perrot et al., [20]	1	-	-	-	-	-	1	-	-	-	-	-	-	-	-	-	-	-
Ribero et al., [21]	1	-	-	-	1	-	-	-	-	-	-	-	-	-	1	1		-
Pertusi et al., [25]	8	2	-	-	2	-	-	2	-	1	5	-	2	1	1	-	1	-
Álvarez-Chinchilla et al., [28]	11	-	-	-	-	-	11	-	-	-	-	-	-	-	-	-	-	-
Mazzella et al., [22]	2	-	1	-	-	-	-	-	-	-	-	-	1	-	-	-	-	-
Paganelli et al., [24]	1	-	1	-	-	-	-	-	-	-	-	-	1	-	-	-	-	-
Avilés-Izquierdo et al., [8]	150	-	37	-	34	42	37	-	-	-	-	-	37	53	51	48	15	13
Kostaki et al., [26]	42	-	-	-	-	-	13	12	-	7	-	-	-	-	-	-	-	-
Simionescu et al., [9]	715	42	66	139	162	71	-	-	-	-	137	-	46	-	-	-	-	-
Tiodorovic et al., [10]	158	-	-	104	-	-	-	-	-	-	-	-	-	-	-	-	-	2

* globular, non-globular and nevus-like patterns were included.

**Table 2:** Dermoscopic Features of Vascular Structures of CMM Reported in Included Studies.

	Vessels Dermoscopic Structures									
	Linear	Glomerular	Dotted	Serpentine	Milky Red Areas	Hairpin	Corkscrew-Winding	Arborizing	Curved	Two or More Vascular Patterns
Bono et al., [5]	-	-	-	-	-	-	34	-	-	25
Virgili et al., [14]	1	-	-	-	-	-	-	-	-	-
Minagawa et al., [15]	-	-	-	-	-	-	-	-	-	1
Jaimes et al., [17]	-	14	7	21	2	11	9	8	-	2
Costa et al., [11]	-	-	-	-	-	-	5	1	-	7
Chernoff et al., [27]	-	-	-	-	-	-	-	-	-	1
Duman et al., [18]	1	-	-	-	-	1		-	1	-
Hoelt et al., [19]	-	-	-	-	1	-	1	-	-	1
Pertusi et. al., [25]	3	-	1	-	-	-	-	1	-	1
Avilés-Izquierdo et al., [8]	40	18	15	9	7	6	3	3	-	50
Kostaki et al., [26]	-	-	-	-	-	-	4	-	-	2
Simionescu et al., [9]	18	-	33	2	1	8	5	2	10	-
Tiodorovic et al., [10]	-	-	-	104	-	-	-	-	-	-

## Data Availability

All data are available within the text.

## Supplementary Materials

The following supporting information can be downloaded at https://www.mdpi.com/article/10.3390/diagnostics16050738/s1: Table S1: Demographic Characteristics of Patients with Cutaneous Melanoma Metastases Across Reported Studies.
