# Supplementary material for: Dermoscopy of Cutaneous Melanoma Metastases: A Comprehensive Literature Review
Source: Diagnostics (Basel). 2026 Mar 2;16(5):738. doi: 10.3390/diagnostics16050738 (PMC12984486; doi:10.3390/diagnostics16050738)
Supplement: Supplementary file 1 [file diagnostics-16-00738-s001.zip › diagnostics-4111400-supplementary.pdf]

Supplementary Table S1. Demographic Characteristics of Patients with Cutaneous Melanoma Metastases Across Reported Studies

	**Number of patients (N=774)**	**Male, n (%) (N=428)**	**Female, n (%) (N=341)**	**Gender not reported, n (%) (N=5)**
Pizzichetta et al., 2002	1	0	1	0
Bono et al., 2004	32	15 (46.9)	17 (53.1)	0
Virgili et al., 2004	1	0	1	0
Minagawa et al., 2009	1	1	0	0
Contreras-Steyls et al., 2011	1	1	0	0
Jaimes et al., 2012	18	12 (66.7)	6 (33.3)	0
Costa et al., 2013	42	20 (47.6)	22 (52.4)	0
Chernoff et al., 2014	1	0	0	1
Duman et al., 2014	1	0	1	0
Hoelt et al., 2014	3	1 (33.3)	2 (66.7)	0
Perrot et al., 2016	1	0	1	0
Ribero et al., 2016	1	0	1	0
Pertusi et al., 2017	1	0	1 (100)	0
Álvarez-Chinchilla et al., 2018	1	1	0	0
Mazzella et al., 2018	2	2	0	0
Paganelli et al., 2018	1	1	0	0
Avilés-Izquierdo et al., 2019	40	21 (52)	19 (48)	0
Kostaki et al., 2022	42	20	22	0

Simionescu et al., 2024	1	1	0	0
Tiodorovic et al., 2024	583	332 (56.9)	247 (42.4)	4 (0.7)
